# Hybrid Nanoparticles as a Novel Tool for Regulating Psychosine-Induced Neuroinflammation and Demyelination In Vitro and Ex vivo

**DOI:** 10.1007/s13311-021-01109-3

**Published:** 2021-09-03

**Authors:** Adryana Clementino, Maria Velasco-Estevez, Francesca Buttini, Fabio Sonvico, Kumlesh K. Dev

**Affiliations:** 1 Drug Development Group, School of Medicine, Trinity College Dublin, Dublin, Ireland; 2 Department of Food and Drug, Università Degli Studi Di Parma, Parma, Italy; 3 National Council for Scientific and Technological Development–CNPq, Brasilia, Brazil; 4 H12O-CNIO Haematological Malignancies Clinical Research Unit, Centro Nacional de Investigaciones Oncológicas (CNIO), Madrid, Spain

**Keywords:** Brain, Neuroinflammation, Neurodegeneration, Nanoparticles, Psychosine, Demyelination

## Abstract

Polymeric nanoparticles are being extensively investigated as an approach for brain delivery of drugs, especially for their controlled release and targeting capacity. Nose-to-brain administration of nanoparticles, bypassing the blood brain barrier, offers a promising strategy to deliver drugs to the central nervous system. Here, we investigated the potential of hybrid nanoparticles as a therapeutic approach for demyelinating diseases, more specifically for Krabbe’s disease. This rare leukodystrophy is characterized by the lack of enzyme galactosylceramidase, leading to the accumulation of toxic psychosine in glial cells causing neuroinflammation, extensive demyelination and death. We present evidence that lecithin/chitosan nanoparticles prevent damage associated with psychosine by sequestering the neurotoxic sphingolipid via physicochemical hydrophobic interactions. We showed how nanoparticles prevented the cytotoxicity caused by psychosine in cultured human astrocytes in vitro, and how the nanoparticle size and PDI augmented while the electrostatic charges of the surface decreased, suggesting a direct interaction between psychosine and the nanoparticles. Moreover, we studied the effects of nanoparticles ex vivo using mouse cerebellar organotypic cultures, observing that nanoparticles prevented the demyelination and axonal damage caused by psychosine, as well as a moderate prevention of the astrocytic death. Taken together, these results suggest that lecithin-chitosan nanoparticles are a potential novel delivery system for drugs for certain demyelinating conditions such as Krabbe’s disease, due to their dual effect: not only are they an efficient platform for drug delivery, but they exert a protective effect themselves in tampering the levels of psychosine accumulation.

**Supplementary Information:**

The online version contains supplementary material available at 10.1007/s13311-021-01109-3.

## Introduction

Krabbe’s disease (KD) or globoid cell leukodystrophy (GLD) is an autosomal recessive pathology caused by genetic defects in the lysosomal enzyme galactosylceramidase (GALC) [1]. This GALC deficiency results in the accumulation of the toxic intermediate metabolite galactosylsphingosine, also called psychosine (Psy) [2]. This excess of psychosine leads to its escape from lysosomes and the formation of aggregates that have cytotoxic effects by various mechanisms, such as mitochondrial dysfunction [3], S1P signaling [4], PKC, JNK and NFkB signaling [3] and inflammatory responses [5]. Ultimately, psychosine accumulation causes demyelination and almost complete loss of the oligodendrocytes in the white matter alongside glial reactivity and infiltration of multinucleated macrophages called ‘globoid cells’ [1, 6]. To date, there is no current treatment for KD, likely in part because of the yet-unknown full mechanism of action of Psy in the pathology, and in part because of a lack of drugs that efficiently reach the CNS and treat the pathophysiological causes of the disease, instead of treating the symptoms.

Despite the important advances in neuroscience in the past years, delivery of drugs to the CNS in an efficient manner still remains a challenge. The major reason for this is the protection that the blood brain barrier (BBB) offers, preventing the access to CNS of a huge number of drugs, especially hydrophilic and large molecular weight compounds [7, 8]. Many drugs cannot cross the BBB efficiently when delivered by the oral or intravenous route; hence, they need to be delivered via more invasive methods, such as hyperosmotic solutions disturbing the BBB permeability or infusion of drugs directly into brain structures [9]. Many of those approaches are either risky or laden with unacceptable side effects, thus leading to the need of developing new platforms for drug deliverable to exploit alternative administration routes into the CNS which are effective and non-invasive.

The development of the pharmaceutical nanotechnology in the recent years has proven nanoparticles to be an effective method for brain delivery of numerous compounds, including drugs for cancer [10], Alzheimer’s disease [11], cerebral ischemia [12] and schizophrenia [13]. Previous studies in our lab have shown the potential of hybrid nanoparticles as an efficient drug delivery system for brain delivery of drugs [14]. Here, we investigated the potential use of hybrid lecithin/chitosan nanoparticles in the treatment of KD due to its protective effects in preventing the effects of psychosine both in vitro and ex vivo.

## Materials and Methods

### Materials and Reagents

Chitosan with deacetylation degree of 95% and viscosity 45 cP was purchased from Primex (batch TM1874, Chitoclear FG, Siglufjordur, Iceland). Lecithin Lipoid^®^ S45 was supplied by Lipoid AD (lot #8,030,760, Ludwigshafen, Germany). Pharmaceutical grade oil vehicles Maisine™ 35–1 (batch 134,565, glyceryl monolinoleate) and Labrafac™ Lipophile WL 1349 (batch 151,738, medium chain triglycerides) were a kind gift from Gatefossé (Saint-Priest, France). Ultrapure water (Purelab Flex, ELGA-Veolia LabWater, High Wycombe, UK) was used for nanoparticles production. All other reagents used for nanoparticles characterization were of analytical grade.

Galactosylsphingosine or psychosine (Psy; sc-202781, batch #C2015, Santa Cruz Biotechnology, Santa Cruz, CA, USA) was prepared as 10 mM stock solution in dimethyl sulfoxide (DMSO; D8418, Sigma-Aldrich, St. Louis, MO, USA). To avoid any cytotoxic effects, organic solvent concentrations during cell treatments were maintained below 0.01%.

### Nanoparticles Preparation and Characterization

Lecithin/chitosan nanoparticles (LCN) were prepared by self-assembly method as previously described [14, 15]. Briefly, 1% w/v chitosan was prepared by dissolving chitosan powder in 0.1 N HCl solution overnight. The same procedure was adopted to produce nanoparticles in organic phase, consisting of 2.5% soybean lecithin dissolved in pure ethanol containing 20 mg of glyceryl monolinoleate and medium chain triglyceride oils. Afterwards, 0.8 ml of ethanol-oily solution of lecithin was added to 10 ml of 0.01% w/v of chitosan aqueous solution under constant agitation of magnetic stirring at 300 rpm and constant temperature of 50 °C. Freshly prepared LCN formulation was kept in agitation and at the same temperature for approximately 10 min to allow complete evaporation of ethanol. LCN particle size and polydispersity index (PDI) were then characterized by dynamic light scattering (DLS), as well as determining the nanoparticle zeta potential by electrophoresing mobility applying phase analysis light scattering (PALS) (Zetasizer Nano ZS, Malvern Instriuments Ltd., Malvern, UK). A chitosan aqueous solution (0.01% w/v) and lecithin dispersion (0.2% w/v) in water were also prepared to be used as controls, following the same manufacturing procedure, but omitting all the other LCN ingredients from the preparation.

### Cryo-Transmission Electro Microscopy (CryoTEM)

For the cryo-TEM, samples of Psy 200 µM, LCN 10 nM and Psy 200 µM + LCN 10 nM were prepared in ultrapure water.

Briefly, 3 μl of sample were applied to Quantifoil holey carbon grids (copper Multi A, Quantifoil Micro Tools GmbH, Jena, Germany). Excess fluid was blotted from the grid for ~ 3 s with Whatman filter paper and then plunge frozen in liquid ethane using a home-made plunge freezer to achieve sample vitrification. Frozen samples were stored in liquid nitrogen until EM imaging. Vitrified samples were imaged using a CM200 FEG transmission EM (FEI, Eindhoven, the Netherlands) operated at 200 keV and equipped with a F224HD 2048 × 2048 CCD camera (TVIPS, Gauting, Germany). EM images were acquired at 50,000× magnification (pixel size 0.331 nm).

### Human Astrocyte Culture

Human astrocytes from foetal cerebral cortex were purchased from ScienCell Research Laboratories (Cat No. #1800, Lot No. 9063 and 11,065; Sciencell Research Laboratories, Carlsbad, CA, USA). Ethics for obtaining human tissue were strictly adhered to by the supplier and complied with local, state and federal laws and regulations, and procedures for using human cells were carefully followed as previously described [16, 17]. Human astrocytes were grown in T75 culture flasks (Corning Inc., Corning, NY, USA) at 37 °C, 95% humidity and 5% CO_2_ using Dulbecco’s Modified Eagle Medium/Nutrient Mixture F-12 (DMEM/F12) culture medium (Hyclone, SH30023, GE Healthcare Life Sciences, Chicago, IL, USA) supplemented with 10% foetal bovine serum (FBS; FB-1090, Labtech, Heathfield, UK), 1% astrocyte growth supplement (AGS; #1852, ScienCell Research Laboratories) and 1% penicillin/streptomycin (P/S; P4333, Sigma Aldrich). Treatments were performed in serum-free DMEM/F12 media supplemented with 1% P/S and cells were serum starved overnight prior treatment.

### Cell Viability Assay

Cell viability was tested using the MTT assay. Briefly, cells were seeded at a density of 120,000 cells/well in a 24-well plate, grown for 48 h and then serum starved overnight prior treatment (4 h incubation). Dose–response curves of nanoparticles were performed to analyse their toxicity (LCN 0.01–2 nM). Similarly, Psy (20 µM) was used to evaluate the efficacy of the treatment (LCN 0.01, 0.1, 1 nM). For comparison purposes, astrocytes were further treated with Psy in presence of the main LCN components, *i.e.* chitosan and lecithin, at concentrations matching those present in 1 nM LCN. Prior to MTT incubation after treatment, representative images of cells in all treatment studies were taken using a CKX41 Olympus inverted microscope (Mason Technologies, Dublin, Ireland). Cells were incubated with 1 mg/ml MTT reagent (M6494, Invitrogen, Thermo Fisher Scientific, Waltham, MA, USA) for 3 h at 37 °C, following the addition of DMSO and absorbance read at 540 nm using an Epoch microplate spectrophotometer (BioTek, Winooski, VE, USA). Absorbance values were directly correlated with the cellular viability and values were calculated as percentage of control.

### Immunocytochemistry

Immunocytochemistry of cultured human astrocytes was performed as previously reported [16, 18]. Briefly, cells were seeded and grown in sterile glass coverslips on 24-well plates and treated as described above (4 h incubation with Psy 20 µM or Psy 20 µM + LCN 1 nM; untreated astrocytes were used as controls). After treatment, cells were washed and fixed in 4% PFA for 5 min on ice. Cells were then washed in PBS and permeabilized with 0.1% Triton-X100 in PBS for 5 min, room temperature (RT). Afterwards, cells were blocked with 1% BSA in PBS + 0.1% Triton-X100, at 4 °C overnight. Following blocking, samples were incubated with primary antibody mouse anti-vimentin (sc373717, Santa Cruz Biotechnology; dilution 1:800) and with secondary antibody Dylight 549 anti-mouse (#715–505-020, Jackson ImmunoResearch, Ely, UK; dilution 1:1000). Cells were when washed and incubated with DAPI (#62,248, Thermo Fisher Scientific; dilution 1:500) for nuclear staining and mounted on a microscope slide using antifade reagent (S36936, Thermo Fisher Scientific). Coverslips were sealed and samples were stored at 4 °C until imaged using an Olympus Bx51 upright fluorescence microscope at 20× magnitude. Image acquisition settings were kept constant across all treatments. Image analysis of fluorescence was made using the ImageJ software version 1.52a (https://imagej.nih.gov/ij).

### Enzyme-Linked Immunosorbent Assay (ELISA)

Human cytokine IL-6 supernatant levels were measured using an IL-6 ELISA Kit (DY206, R&D Systems, Minneapolis, MN, USA), following manufacturer’s instructions. Briefly, 96-well plates of maxiabsorbant (#442,404, SigmaAldrich) were coated with the capture antibody diluted in PBS, overnight at 4 °C. Plates were washed in 0.05% PBS/Tween-20 and blocked at RT for a minimum of 1 h. Plates were then washed again and incubated with samples and standards for 2 h at RT. After following washes, plates were incubated with detection antibody for further 2 h at RT and washed again before incubation with streptavidin-HRP solution for 20 min in the dark. ELISA was developed using substrate solution (DY999, R&D Systems). The reaction was stopped by adding 2 N H_2_SO_4_ and absorbance was read at 450 nm with wavelength correction at 570 nm.

### Cerebellar Organotypic Slice Culture

Mouse cerebellar organotypic slice culture was performed using postnatal day 10 C57bl/6mice (P10) provided by BioResources Unit, Trinity College Dublin (Ireland). All tissue was isolated in accordance with EU regulations and internal protocols approved by Trinity College Dublin ethical committee. Mice were sacrificed by decapitation, the skull removed and the cerebellum separated from hindbrain. Cerebellum was cut into 400 μm parasagittal sections using a McIlwan tissue chopper. Tissue was placed into Opti-MEM (#31,985, Gibco, Thermo Fisher Scientific) and separated into individual slices under a dissection microscope. Five slices were placed per cell culture insert (PICMORG50, Merk Millipore, Burlington, MA, USA) and grown at 35.5 °C, 95% humidity and 5% CO_2_. Slices were grown for the first 4 days in media containing 50% Opti-MEM, 25% Hank’s buffered salt solution (HBSS; #14,025–050, Gibco, Thermo Fisher Scientific) and 25% heat-inactivated horse serum (#26,050–088, Gibco, Thermo Fisher Scientific) supplemented with 2 mM Glutamax (#35,050, Gibco, Thermo Fisher Scientific), 28 mM D-Glucose (G8769, SigmaAldrich), 1% P/S and HEPES (#15,630–056, Gibco, Thermo Fisher Scientific), changing the media at day in vitro 1 (DIV1) and DIV4. At DIV7, media was changed to a serum-free media containing 96% Neurobasal-A (#10,888–022, Gibco, Thermo Fisher Scientific) and 2% B-27 supplement (#17,504–044, Gibco, Thermo Fisher Scientific) supplemented with 1% P/S, 28 mM D-Glucose, 2 mM Glutamax and 10 mM HEPES. Media was changed again at DIV10 and treatment was performed at DIV12.

### Immunofluorescence

After treatment of organotypic cultures, fixation was done by increasing concentration of PFA (1%, 2%, 3% and 4%) for 5 min each. Blocking and permeabilization were performed overnight at 4 °C in blocking solution 10% bovine serum albumin (BSA; #1,073,508,600, SigmaAldrich) in PBS + 0.5% Triton-X100 (Tx). Primary antibodies were diluted in 2% BSA in PBS + 0.1% Tx, incubating for 48 h at 4 °C. Slices were then washed in PBS 5 times for 5 min and incubated with secondary antibodies diluted in 2% BSA + 0.1% Tx overnight at 4 °C. Slices were then washed again and mounted on a slide microscope using SlowFade^®^ Gold antifade reagent (S36936, Life Technologies, Thermo Fischer Scientific).

### Confocal Microscopy and Image Analysis

Imaging of organotypic cultures was performed using a Leica SP8 confocal microscope (Leica Microsystems, Wetzlar, Germany) in TBSI, Trinity College Dublin. Five slices per condition were grown and an average of 25–3 images per condition were captured at 20 × magnification. The images were exported as 8-bit.tif files for analysis using the software package ImageJ by FIJI (https://imagej.net/Fiji). Intensity values were normalized to the average of control for each experiment and each marker. To analyse the expression of SMI-32 in the white matter tracts (WMT), the software package Imaris^®^ (http://www.bitplane.com/imaris/imaris) was used. Briefly, the WMT on each image were selected and the area of SMI-32 immunoreactivity was measured.

### Statistical Analysis

All statistical analysis was performed using GraphPad Prism 8 (GraphPad^®^ Software, San Diego, CA, USA). Assessment of the normality of the data was carried out by column statistics with D’Agostino analysis before any further statistical test was performed. To analyse cell viability via MTT assay, immunocytochemistry images and IL-6 cytokine release under multiple conditions, repeated measures one-way ANOVA test was performed, as data in every experiment was matched and with Gaussian distribution. Holm-Sidak multiple comparisons post hoc test was run in conjunction with one-way ANOVA and all groups were compared between themselves. Similarly, to analyse fluorescence intensity levels in organotypic slice cultures, repeated measures one-way ANOVA following Sidak’s post hoc test was performed. *P* values < 0.05 were considered statistically significant for all experiments. Data is presented as mean ± standard error of the mean (SEM). Further details of the statistical analysis performed are given in each figure legend and the results section.

## Results

### Hybrid Lecithin/Chitosan Nanoparticles Protect Human Astrocytes from Psychosine-Induced Cytotoxicity In Vitro

**Fig. 1:**
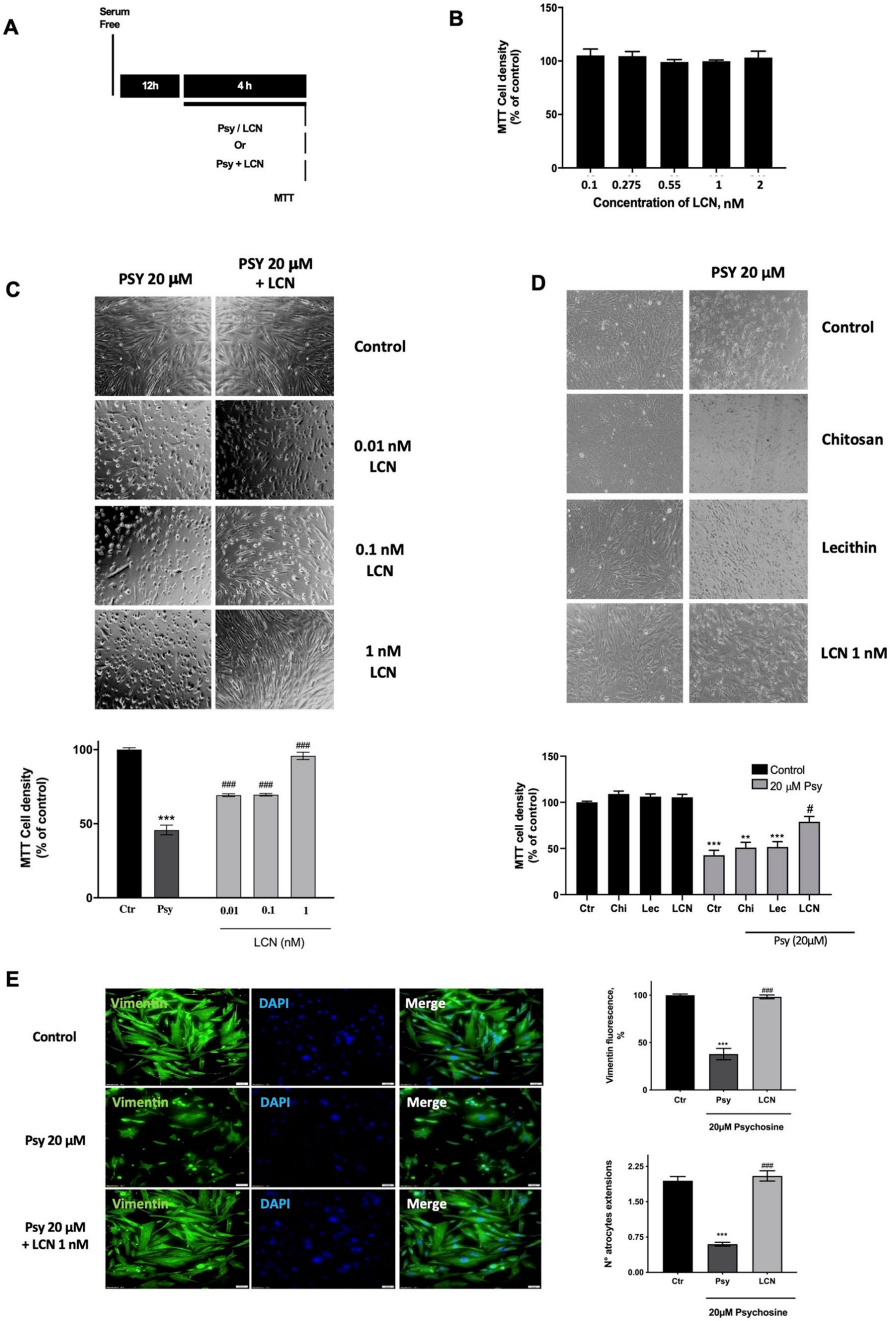
Nanoparticles (but not the individual components) protect from Psy-induced astrocytic cell death. **A** Experimental diagram of treatments and analysis. Briefly, human astrocytes were serum starved overnight and then treated for 4 h with psychosine (20 µM, Psy), lecithin/chitosan nanoparticles (LCN, 0.1–2 nM) or the combination of Psy (20 µM) and increasing concentrations of LCN (0.01, 0.1 and 1 nM). **B** LCN nanoparticles did not show cytotoxicity in human astrocytes at any of the concentration used, as measured by MTT assay and having untreated astrocytes as control. **C** Light microscope images and MTT assay showed LCN protection against 20 µM Psy-induced cell death, in a dose-dependent manner, tested at 0.01 nM, 0.1 nM and 1 nM. **D** However, light microscopy images and MTT assay showed that lecithin or chitosan on their own failed to protect astrocytes from Psy effects. **E** Fluorescent images at 20× magnification of human astrocytes labelled for vimentin (green) and DAPI (blue) showing a protective effect of LCN from the Psy-induced toxic effects, measured by intensity of vimentin expression and number of astrocytic extensions. Scale bar = 50 µm. Fluorescence analysis was performed using ImageJ software. Data shown as mean ± SEM. Statistical analysis performed using one-way ANOVA following Holm-Sidak’s post hoc test. ***p < 0.001 compared to control; ^###^*p* < 0.001 compared to Psy. *N* = 8 for cell viability assays; *N* = 4 for fluorescence analysis

Administering drugs encapsulated into polymeric nanoparticles has proven to be an efficient way of targeting the CNS and improving their pharmacokinetics. Here, we developed and studied hybrid LCN which can potentially be used to encapsulate drugs for brain delivery after intranasal administration. Following a simple and reproducible manufacturing technique which exploits the electrostatic self-assembly of phospholipids and polysaccharides, LCN were obtained with relatively small particle size (234.2 ± 8.8 nm), presenting narrow particle size distribution (PDI 0.094 ± 0.009) and surface charge sufficiently strong to provide good physical stability (zeta potential of + 40.21 ± 1.09 mV). Here, we first tested the toxicity of blank LCN at increasing concentrations in cultured human astrocytes. LCN were incubated for 6 h at concentrations ranging from 0.1 to 2 nM, and cell viability of astrocytes was measured by MTT assay. We observed that none of the LCN concentrations used evidenced significant cytotoxic effects on the astrocytes (Fig. 1B). We then tested the potential protection of these LCN against psychosine toxicity. Psychosine is a toxin that accumulates in the brains of patients affected by Krabbe’s disease, due to the deficit of the lysosomal enzyme galactocerebrosidase [6]. Previous studies in our lab have shown that Psy induces astrocytic cell death in a concentration and time-dependent manner [4, 19]. Thus, astrocytes were incubated with 20 µM of Psy in the presence or absence of LCN at concentrations ranging from 0.01 to 1 nM. It was observed that LCN efficiently protect against Psy-induced cytotoxicity, with the highest protection observed at 1 nM LCN, presenting cell densities similar to control (LCN 0.01 nM: 69.34% ± 2.98%; LCN 0.1 nM: 69.59% ± 2.8%; LCN 1 nM: 95.80% ± 4.86% of control vs Psy: 45.73% ± 6.63%) (Fig. 1C). In order to better understand the mechanism of action through which LCN protect from Psy-induced toxicity, we analysed the effects on cell viability of the individual components of LCN, *i.e.* lecithin and chitosan, at concentrations matching those present in LCN 1 nM. Interestingly, the individual components of the nanoparticles had no protective effects on astrocytes (chitosan: 50.89% ± 5.79; lecithin: 51.74% ± 5.69 vs Psy: 42.71% ± 5.30%) (Fig. 1D). To further assess the protective effects of LCN, we analysed the effects of LCN on the expression of the astrocytic cytoskeleton marker vimentin by immunofluorescence. Treatment of human astrocytes with 20 µM Psy caused a decrease in the expression of vimentin in astrocytes and in the number of astrocytic projections (Psy: 37.86 ± 6.02% vimentin expression compared to control; 0.24 ± 0.08 astrocytic projections vs 2.14 ± 0.16 for control). This decrease, however, was prevented by 1 nM LCN co-treatment, where values of vimentin fluorescence intensity and number of projections were similar to control conditions (98.30 ± 2.4% vimentin expression and 2.05 ± 0.11 astrocytic projections, respectively) (Fig. 1E).

### Nanoparticles Effects are not Mediated via Inflammatory Mechanisms

**Fig. 2:**
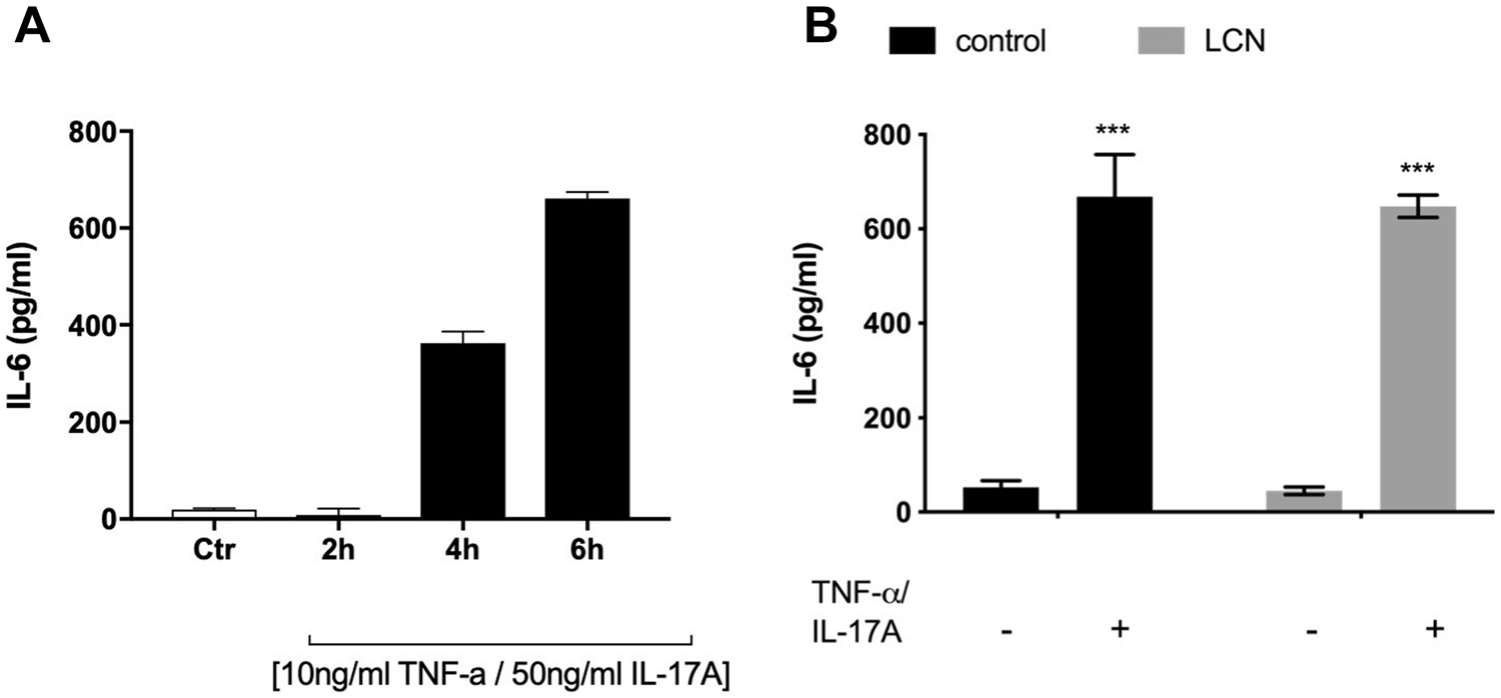
Nanoparticles effects are not mediated via inflammatory mechanism. **A** TNFα and IL-17a increased the level of IL-6 release in human astrocytes. Human astrocytes were seeded at 3.9 × 10^5^ cells/ml confluency and grown for 48 h. Cells were then treated with 10 ng/ml TNF-α and 50 ng/ml IL-17a for 2 h, 4 h and 6 h. Graph shows the secretion of IL-6 over time under cytokine stimuli. **B** LCN nanoparticles did not affect IL-6 release in human astrocytes after TNFα/IL-17a stimulation. Data shown as mean ± SEM. Repeated measures one-way ANOVA following Holm-Sidak’s post hoc test was performed. ***p < 0.001 compared to negative control. *N* = 6

Previous studies in our lab have shown that human astrocytes in vitro express IL-17A receptor and that stimulation of this receptor causes the release of proinflammatory cytokine IL-6, which is synergistically increased by the addition of TNFα [18], where maximum release of IL-6 is observed at concentrations of 10 ng/ml TNFα plus 50 ng/ml IL-17a for 18 h sphinganine glycosylated derivatives*.* Here, we confirm this proinflammatory effect at 2, 4 and 6 h, showing that TNFα + IL-17a lead to the release of IL-6 after 4 h of treatment, with maximum release at 6 h treatment (661.3 ± 22.6 pg/ml) (Fig. 2A). To analyse whether the protective effects of LCN were restricted to preventing the cytotoxic effects of Psy or they could protect against proinflammatory stimuli as well, human astrocytes were stimulated with 10 ng/ml TNFα plus 50 ng/ml IL-17a in the absence or presence of 1 nM LCN for 6 h. It was observed that while LCN did not cause any release of IL-6 on their own (45.0 ± 7.7 pg/ml vs control conditions of 52.17 ± 14.67 pg/ml), they failed to prevent the release of IL-6 caused by TNFα + IL-17a (648.0 ± 23.5 pg/ml vs 668.2 ± 89.5 g/ml) (Fig. 2B). This suggests that the protective effects of LCN are restricted to preventing the damage to astrocytes induced by Psy but not those caused by the inflammatory responses.

### LCN-Protective Effect is Based on their Structure

**Fig. 3:**
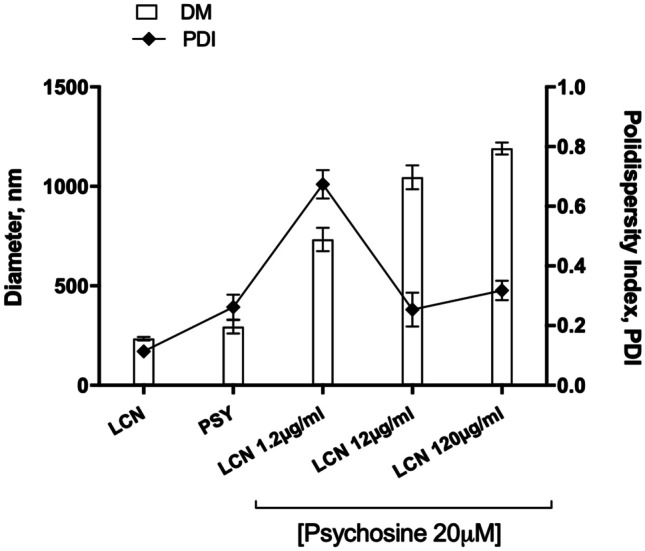
Evidence of mechanical effect of LCN nanoparticles sequestering Psy. Hydrodynamic particle diameter and particles size distribution of LCN nanoparticles combined with Psy were investigated by DLS measurements. Particles diameter increased proportionally with the crescent concentration of LCN plus 20 µM Psy. Data shown as mean ± SEM. *N* = 3 with six runs per analysis

**Fig. 4:**
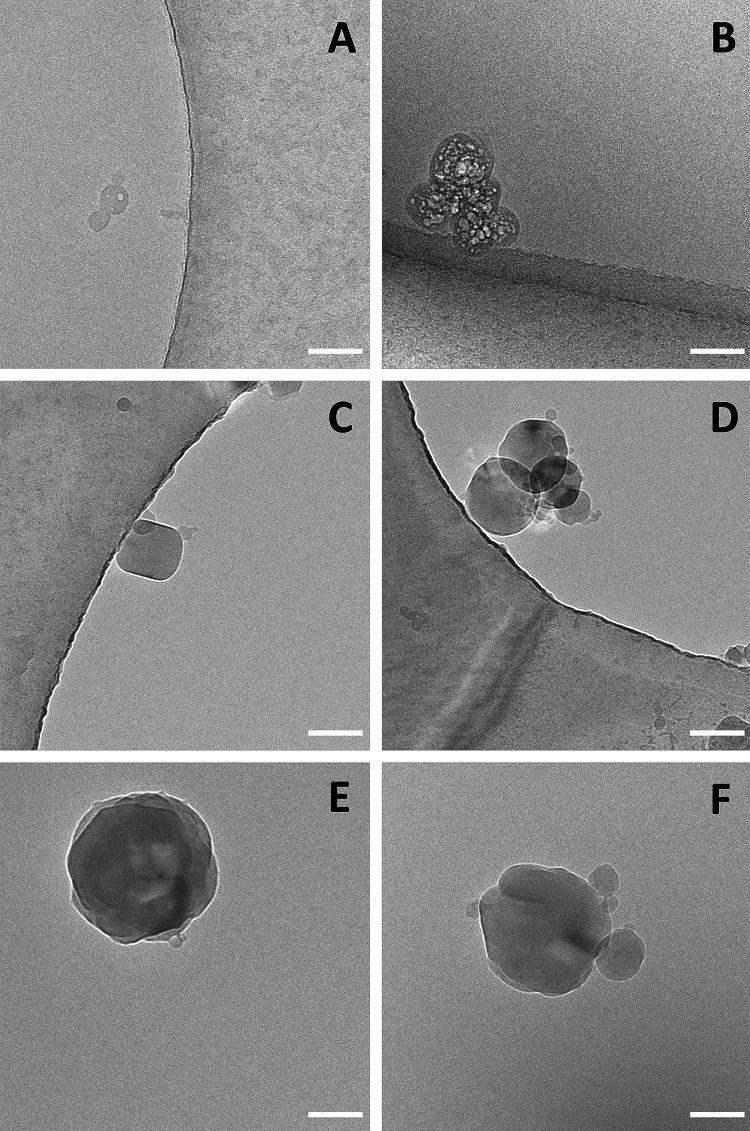
CryoTEM images of Psy, LCN and their combination. Representative cryoTEM images obtained for dilutions in ultrapure water of **A, B** Psy (200 µM); **C, D** LCN nanoparticles (10 nM) and **E, F** their combination (Psy 200 µM + LCN 10 nM). Scale bars = 100 nm

Having observed that assembled LCN protected against cytotoxicity induced by Psy but had no effect on preventing cytokine release caused by TNFα + IL-17a, we hypothesize that this effect could be related on nanoparticles direct interaction with Psy driven by the particle properties. Thus, the physicochemical properties of LCN were analysed using DLS measurements, reporting particle diameter, particle size distribution and zeta potential of LCN at 0.01, 0.1 and 1 nM in the presence of 20 µM of Psy. Interestingly, we observed that in the presence of Psy, LCN were found to have a concentration-dependent increase in their average diameter, compared to LCN alone (Psy + LCN 0.01 nM: 733.1 ± 58.3 nm; Psy + LCN 0.1 nM: 1046 ± 60.4 nm; Psy + LCN 1 nM: 1191 ± 30.2 nm vs control LCN: 234.2 ± 8.8 nm), while at the same time they decreased their surface charge (Psy + LCN 0.01 nM: + 3.20 ± 0.87 mV; Psy + LCN 0.1 nM: + 1.09 ± 0.04 mV; Psy + LCN 1 nM: + 0.69 ± 0.02 mV). Furthermore, PDI of LCN increased in the presence of Psy (Psy + LCN 0.01 nM: 0.684 ± 0.082; Psy + LCN 0.1 nM: 0.321 ± 0.056; Psy + LCN 1 nM: 0.218 ± 0.048 nm vs control LCN: 0.094 ± 0.009) (Fig. 3) indicating a broad particle size distribution. These results suggest that LCN physical interactions with Psy may be the responsible for the protective effects observed in vitro on cultured astrocytes. Since, however, also Psy by itself at this concentration forms micelles with a low positive surface charge and aggregates (294.5 ± 33.5 nm, PDI 0.262 ± 0.072 and zeta potential 9.9 ± 0.2 mV; see also Fig. 3), as reported also by others [20], we decided to proceed with the direct observation of the samples by cryoTEM. In the cryoTEM images, it appears clear that Psy forms small structures in the range of 35–50 nm attributed to micelles (Fig. 4A) but also larger agglomerates (120–200 nm) with a more complex inner structure (Fig. 4B). LCN nanoparticles, on the contrary, appear as spherical particles with a wide particle size distrbution (70–200 nm, Fig. 4C) often associated in groups of multiple particles (Fig. 4D). When the two materials were mixed, it was impossible to identify Psy micelles or agglomerates, while the LCN structures were still present but with an important increase of larger structures of around 200–300 nm (Fig. 4E). Smaller structures bound to the surface of larger particles were also observed (Fig. 4F).

### Psy-Induced Demyelination is Prevented by LCN in Organotypic Slice Cultures

**Fig. 5:**
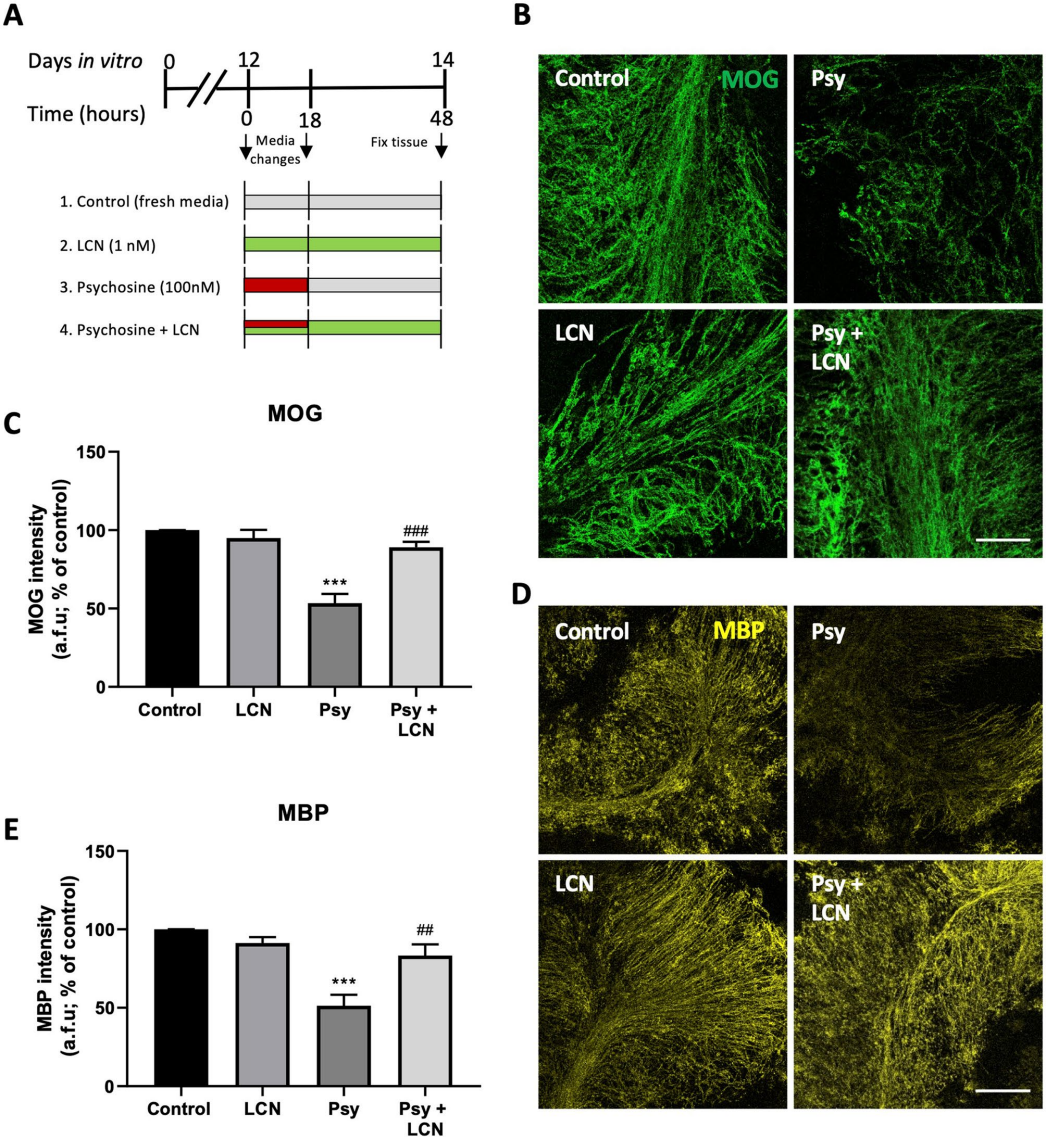
LCN protect against Psy-induced demyelination. Cerebellar organotypic slice cultures were used as an ex vivo model. **A** Schematic treatment protocol, showing that cerebellar slices were obtained from P10 mice and cultures in vitro for 12 days. At DIV12, treatment was added and replaced to fresh change after 18 h, until 48 h treatment. Slices were then fixed, blocked and prepared for staining. **B, C** Analysis of MOG levels show a decrease in the myelin marker under psychosine treatment, which was prevented by LCN. **D, E** Similar results were obtained for MBP levels. Scale bar = 100 µm. Data is shown as mean ± SEM. Repeated measures one-way ANOVA following Holm-Sidak’s post hoc test was performed. *N* = 8

Psy accumulation in Krabbe’s disease leads to oligodendrocyte death and demyelination [2]. We have previously demonstrated in our lab that Psy also induces demyelination in mouse cerebellar organotypic slice culture [4, 19] and that this system can be used as an ex vivo model of demyelinating diseases to study pharmacological effects on demyelination, neuronal and glial cell effects. Organotypic slices from P10 C57Bl/6 mice were grown in vitro for 12 days (DIV12), when they were treated with in the presence or absence of 100 nM Psy with or without 1 nM LCN (Fig. 5A). As previously observed, Psy treatment induced demyelination in slices, reducing the levels of expression of both myelin markers MOG (53.39% ± 5.92% of control values, ***p < 0.001, RM one-way ANOVA Sidak’s multiple comparisons test) (Fig. 5B, C) and MBP (51.35% ± 7.00% of control, ***p < 0.001) (Fig. 5D, E). However, this demyelinating effect was prevented with co-treatment of Psy plus 1 nM LCN (MOG: 89.05% ± 3.462% of control, ^###^*p* < 0.001 compared to Psy; MBP: 83.27% ± 7.19% of control, ^##^*p* < 0.01 compared to Psy) (Fig. 5B–E). These results corroborate the protective effect of LCN preventing the cytotoxic effects of Psy observed in astrocytes in vitro.

### LCN Prevents Psy-Induced Axonal Injury

**Fig. 6:**
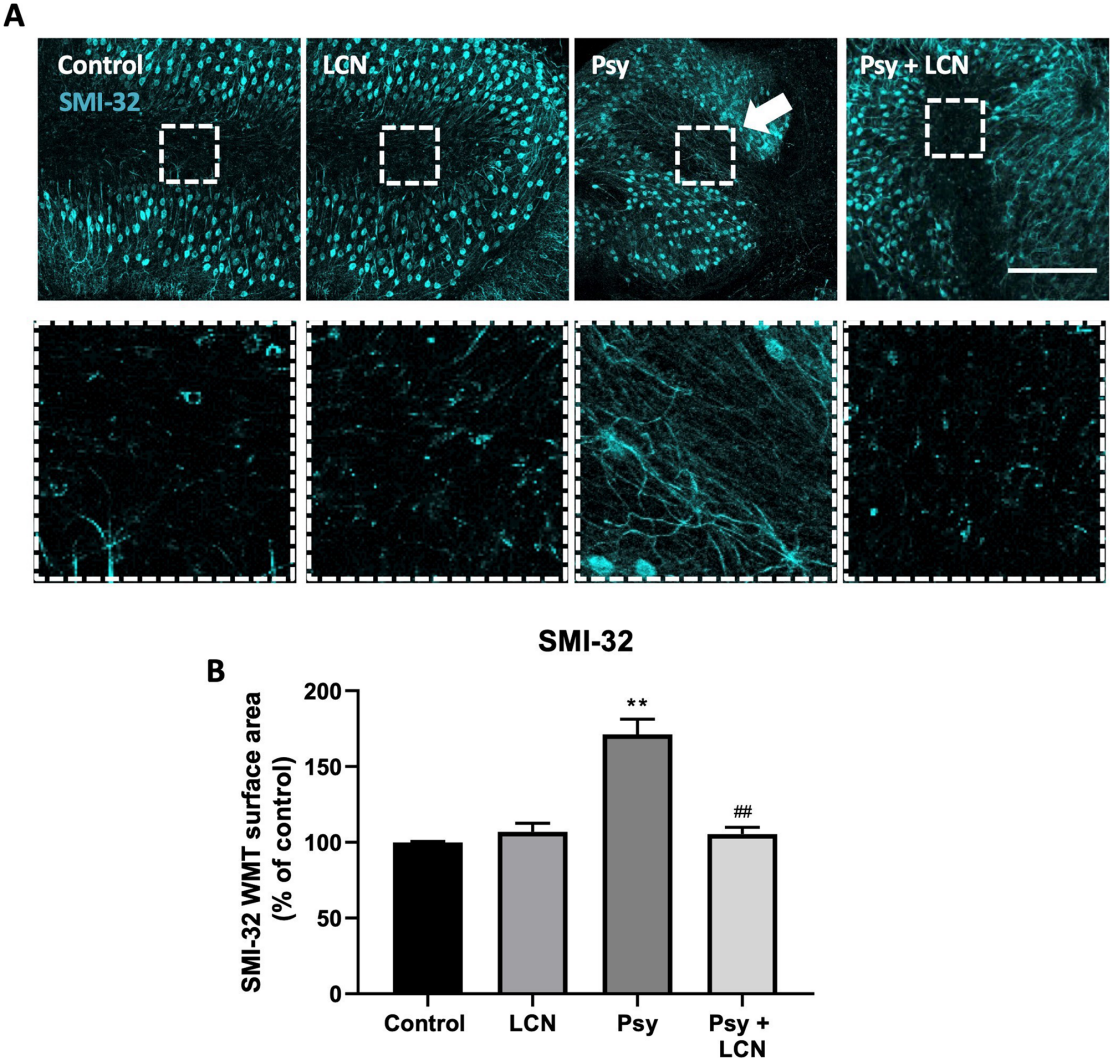
LCN prevented Psy-mediated axonal injury in cerebellar slices. **A** Immunofluorescence analysis of neuronal SMI-32 marker in cerebellar slices. **B** Analysis of the surface expression reveals an increase of axonal SMI-32 in the white matter tracts under psychosine treatment. However, this expression was prevented in co-treatment with LCN. Scale bar = 100 µm. Data shown as mean ± SEM. Repeated measures one-way ANOVA following Holm-Sidak’s post hoc test was performed. *N* = 6

We and others have previously reported axonal injury under demyelinating conditions [19, 21, 22]. Thus, we examined the effects of LCN in the control of axonal damage induced by Psy, by measuring the expression of SMI-32, a non-phosphorylated epitope of NFH [23]. In accordance with our previous studies, Psy induced axonal damage related to demyelination, as observed in the higher expression of SMI-32 in the axonal tracts of the *arbor vitae* (157.3% ± 5.66% of control values, **p < 0.01, RM one-way ANOVA, Sidak’s multiple comparisons test). However, LCN treatment in organotypic slices not only prevented demyelination but also prevented the axonal expression of SMI-32 in the myelinated tracts as well (103.5% ± 5.84%, ^#^*p* < 0.05 compared to Psy) (Fig. 6). This suggests that LCN prevents the Psy-induced demyelination thus preventing the associated axonal damage on the cerebellar Purkinje neurons.

### Astrocytic Death Induced by Psy was not Fully Prevented by LCN Ex Vivo

**Fig. 7:**
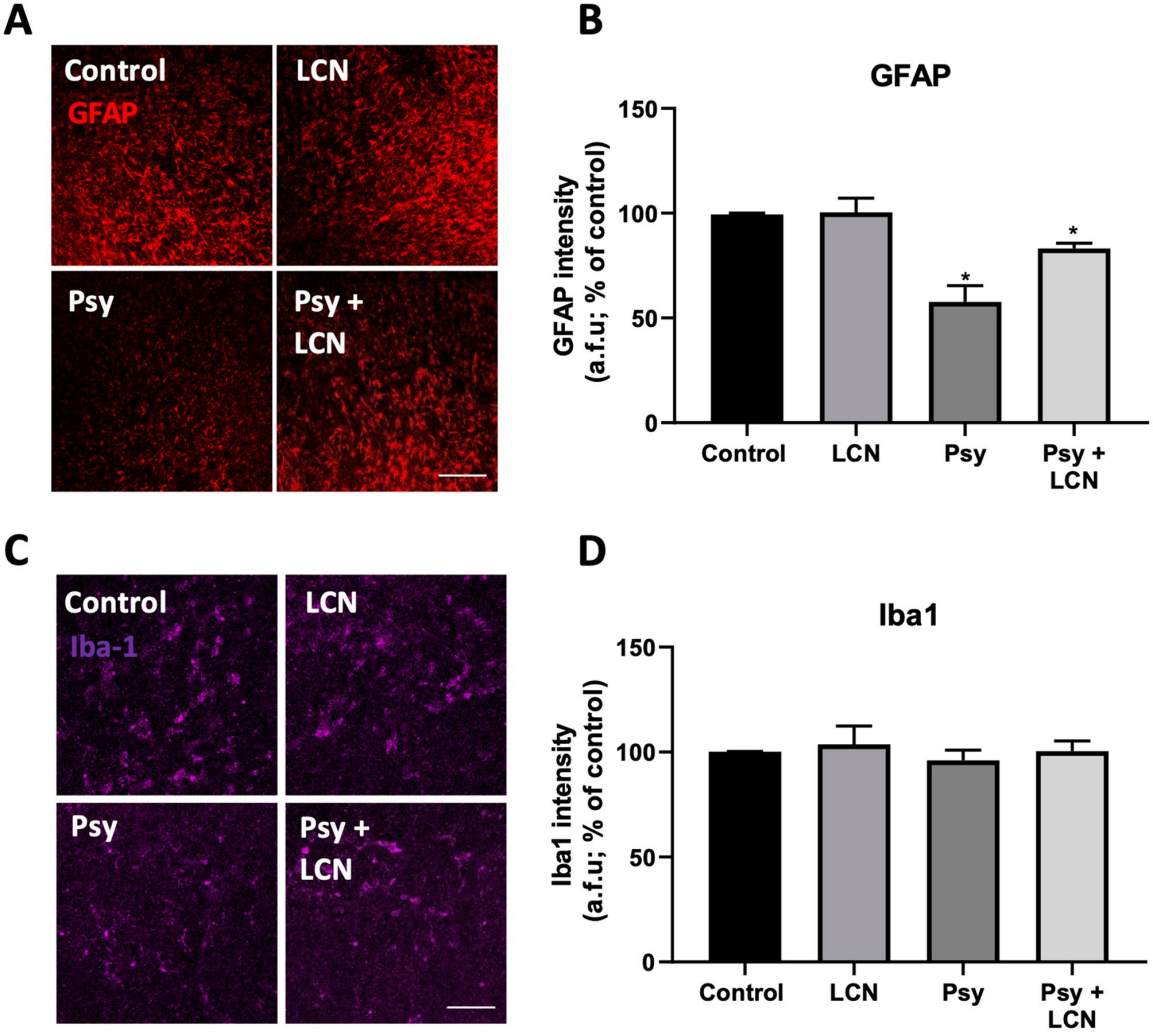
Psy-induced astrocytic death was not fully prevented by LCN in cerebellar slices. **A, B** Immunofluorescence of astrocytic GFAP marker showed a significant decrease of GFAP levels in Psy-treated slices, while LCN had little effect reverting this loss. **C, D** Astrocytic and early glia marker, vimentin, showed no apparent difference in the fluorescence levels of vimentin. **E, F** Analysis of the microglial marker Iba-1 showed no effects on the levels of intensity or apparent morphology of microglia. Scale bars = 100 µm. Data shown as mean ± SEM. Repeated measures one-way ANOVA following Holm-Sidak’s post hoc test was performed. *N* = 6

As we observed a protective effect of LCN preventing the cytotoxicity of Psy in human astrocytes in vitro, we then examined the expression of astrocytic maker GFAP and astrocytic and early astrocytic marker vimentin. Similar to what observed in previous studies in our lab, Psy induced astrocytic death measured by a decrease in the expression of GFAP (57.68% ± 7.71% of control values, *p < 0.05, RM one-way ANOVA with Sidak’s multiple comparisons test) (Fig. 7A, B) and a non-significant tendency to decrease in the expression of vimentin (79.15% ± 8.49%, of control values, n.s.) (Fig. 7C, D). In contrast with what observed in our in vitro experiments (Fig. 1), LCN treatment did not prevent the Psy-induced astrocytic death ex vivo to control levels, although it caused a non-significant increase in the expression of GFAP compared to Psy-treated levels (83.14% ± 2.57%, *p < 0.05 compared to control, n.s. compared to Psy) (Fig. 7A, B). When observing the effects on microglia by measuring the expression of the microglial marker Iba-1, none of the treatments had any apparent effect on the levels of expression of this microglial marker (Fig. 7E, F).

## Discussion

### Summary of Findings

Krabbe’s disease is a fatal leukodystrophy with no current curative treatment. Therefore, a need for new drugs and pharmacological approaches that can improve and cure these patients is urgent. One of the main challenges scientists face when developing new treatments for CNS pathologies is the BBB. Thus, developing new therapeutic approaches that are able to bypass the BBB improving drug delivery to the brain is an ideal solution. Here, we report the potential use of hybrid lecithin/chitosan nanoparticles designed and already studied for nose-to-brain administration [14] in the dampening of psychosine and its protective effects against the cytotoxic effects of this toxin both, in vitro using human astrocytes and ex vivo using organotypic slice cultures. In this study, we have shown that these nanoparticles can prevent Psy-induced demyelination ex vivo, also preventing the associated axonal damage. Furthermore, despite that they failed to fully protect against the astrocytic death caused by Psy in this model, they led to lower cytotoxicity in astrocytes and early glia, in line with the full protection observed in vitro.

### Hybrid Lecithin/Chitosan Nanoparticles as a New Therapeutic Approach for CNS Pathologies

As mentioned before, BBB separates the CNS from the systemic circulation protecting it from the entry of many pathogens and cells, but also being a challenge when delivering drugs to the CNS [7]. It is considered that the nose-to-brain administration route with nanoparticle-based delivery strategies could improve the cerebral bioavailability of many drugs [24, 25]. Recently, our group has proposed that hybrid nanoparticles are composed of natural polysaccharides and phospholipids as an efficient system for drug delivery for various administration routes [14, 26, 27]. In this novel study, we proposed the lecithin/chitosan nanoparticles formed by spontaneous self-assembly not only as an efficient vehicle for drug delivery to the brain, but as a treatment on its own for the rare leukodystrophy Krabbe’s disease model in vitro and ex vivo.

### LCN Protect from Psy-Induced Cytotoxicity Likely due to its Physicochemical Properties

In the past years, astrocytes and their function have gained recognition as an important factor contributing to the onset and progression of many CNS pathologies [28], and it has been shown in multiple studies that they contribute to the neuroinflammatory state of the CNS, thus contributing to chronic and exacerbated inflammation in disease [18, 29]. Previous studies in our lab have also shown that Psy causes dysfunction in astrocytic function and is cytotoxic to this cell type, as well as showing that these effects could be closely linked to the pathogenesis of Krabbe’s disease [4, 19, 30]. Here, we have shown that Psy induces morphological changes in astrocytes in vitro as observed by a reduction in the number of astrocytic projections, as well as cell death as measured by MTT assay (Fig. 1). Interestingly, we observed that the use of blank LCN not only demonstrated to be safe and well tolerated by human astrocytes (biocompatibility already observed for other human cell lines such as nasal RPMI2650 and monocytic THP-1 cells), but more importantly they were effective in protecting against the cytotoxic effects of Psy, without the action of any encapsulated drug. This supports the hypothesis that LCN structure is the responsible for this effect, probably correlated to the composition of nanoparticles formulation and due to its physicochemical properties. Psychosine, as a sphinganine glycosylated derivatives, has a pKa of 7.18. At acidic pHs (~ 4.4), Psy behaves as a mild soluble amphiphile forming micelles; however, at neutral environments, as in Krabbe’s disease where Psy tends to accumulate in extralysosomal spaces, Psy is only partially charged. At pH 7.4, Psy micelles low surface charge may favor its organization into a more compact structure resulting in aggregates, as already observed by others [20]. This is in accordance to results presented here, where Psy alone was found by DLS to form structures with an average diameter of around 300 nm and a slightly positive surface charge (~ 10 mV). Psy micellar structures and larger agglomerates where evidenced in the cryoTEM images (Fig. 4A, B), even if the size of observed structures was significantly smaller compared to DLS data. These discrepancies are common when comparing DLS results with electron microscopy, since the DLS measures the hydrodynamic diameter of colloidal particles and provides an intensity weighed particle size distribution. Thus, the DLS measure is strongly affected by larger particles and agglomerates that scatter light with higher intensity and ‘mask’ the smaller particle scattering (see [31]). When evaluating the effect of the presence of Psy on the size, PDI and surface charge of lecithin/chitosan nanoparticles, we observed that the LCN increased in size while the electrical surface charge decreased, suggesting an interactions of the sphingolipid with the nanoparticles (Fig. 3). CryoTEM images disproved our initial and simplistic supposition that Psy structures were forming agglomerates with LCN nanoparticles by adsorbing on the nanoparticles surface. An alternative hypothesis could be that Psy, as a result of its tensioactive properties, is inducing a reorganization of the LCN, possibly by coalescence of smaller particles.

This hypothesis is supported by some previous observations which evidenced that Psy 50 µM toxicity on cultured primary fibroblasts from GLD patients was reduced by increasing concentrations of hydroxypropyl-β-cyclodextrin, a molecule able to complex hydrophobic molecules [32]. Similarly, Psy has been shown to integrate into phospholipid bilayers disturbing surface electrostatics and disordering the membrane hydrophobic portion [33]. In another study supporting the idea that Psy has mainly a membrane-based toxicity mechanism, it was highlighted that increasing concentrations of Psy (5–50 µM) caused the swelling of DOPC liposomes in consequence of the sphingolipid partitioning in to the phospholipid bilayer [34]. These evidences appear particularly relevant for explaining the interaction of Psy with LCN nanoparticles, where phospholipids constitute the main component acting as surfactant for the lipid core and interacting electrostatically with chitosan. A perturbation in these functions, mediated by Psy integration in the nanoparticle structure, could cause particle instability leading to coalescence to reduce the surface area exposed to the water environment and favouring the attainment of a new equilibrium. Interestingly, however, no protective effect was observed for chitosan or lecithin alone, suggesting that nanoparticle components, as well as their specific organization (phospholipids surrounding a lipid core and stabilized by chitosan), play an important role for their efficient interaction with psychosine. These findings suggest that the interaction of Psy in LCN structure prevents the capacity of Psy to disrupt the astrocytic membrane and/or permeate the cells by physical sequestration, thus inhibiting its cytotoxic effects. How to exploit this scavenging action in a clinically relevant setting requires further investigation.

### Psy-Induced Demyelination and Axonal Damage is Prevented by LCN Treatment Ex Vivo

As previously mentioned, Psy is a metabolic toxin that accumulates in the KD brains and is responsible for the oligodendrocyte death and extensive demyelination of both the central and peripheral nervous systems in pathology, leading to patients’ death after few years of diagnosis [1, 2]. It has been demonstrated that the ex vivo model organotypic slice cultures are a viable method to study potential pharmacological treatments for KD or similar pathologies, as demyelination and Psy-associated effects can be mimic by adding Psy to this ex vivo system [4, 19]. Furthermore, cerebellar organotypic slice cultures provide a challenging environment of LCN, since the culture media will be rich in neurotransmitters, proteins and other substances secreted by cells [30, 35, 36] and eventually competing or preventing Psy from interacting with LCN.

In this study, we have shown that treating organotypic slice cultures with LCN prevents the demyelination observed by decreased level of the myelin markers MBP and MOG (Fig. 5) and a prevention of the associated axonal damage, measured by expression of SMI-32 in the axons of the *arbor vitae* (Fig. 6). These results support our hypothesis that LCN are able to aggregate with Psy preventing its pathological effects, as observed in our ex vivo experiments.

When studying the effects of Psy and LCN on glial cells, we did not observe any change in microglia as measured by the microglia marker Iba-1 (Fig. 6). However, we note that this marker does not provide an insight into microglia reactivity, and therefore, further studies investigating the state of microglia would be required to analysed whether there is microglial activation under Psy conditions and if so, whether LCN are able to revert this effect. On the other hand, when analysing the astrocytic marker GFAP and early glia marker vimentin, we observed that Psy induce astrocytic death as measured by both a significant decrease in levels of GFAP and lower levels of vimentin, although the latter was not statistically significant (Fig. 6). These effects observed under Psy treatment are in line with what previous studies in our lab have shown [19, 22]. As expected, LCN also prevented astrocytic death induced by Psy, although it did not prevent it fully. However, LCN treatment prevented the astrocytic death to a similar extend as our in vitro studies in presence of Psy. This might be because of a higher sensitivity of astrocytes to the toxicity of Psy compared to other cell types as oligodendrocytes, likely due to different mechanisms of action of Psy depending on the cell type. We hypothesize that the protective effects of LCN against Psy are mainly due to physicochemical interactions which prevent Psy from exerting its toxic effects on the cellular systems. However, we also speculate the possibility of a competition between LCN and Psy for the astrocytic phospholipase A2 (PLA2). Previous studies in our lab have shown that activation of PLA2 by Psy leads to the formation of arachidonic acid metabolites, involved in inflammatory processes, oxidative stress and apoptosis in astrocytes [19]. In addition to this, we have also shown that lecithin, the main component of LCN, is a substrate of PLA2 [14, 26, 37]. Thus, a competitive inhibition of PLA2 by the LCN could also contribute to the prevention of Psy toxicity and might explain the differences in the extension of the protective effects of LCN in oligodendrocytes and astrocytes.

## Conclusion

The data presented here demonstrate a novel and promising role of hybrid lecithin/chitosan nanoparticles in the treatment of demyelinating pathologies, specifically Krabbe’s disease pathologies and those with an accumulation of toxins such as psychosine. Here, we report for the first time, the capacity of LCN to prevent Psy-induced glial cell death and demyelination both in vitro and ex vivo. We hypothesized that these effects are mainly relate to the physicochemical interactions of the LCN with the toxins, thus preventing Psy to exert its pathological effects. Therefore, this study gives evidence of the potential use of LCN not only as an efficient platform to delivery drugs to the CNS, as previously reported, but as a treatment itself in demyelinating pathologies such as Krabbe’s disease.

## Supplementary Information

Below is the link to the electronic supplementary material.

Supplementary file1 (PDF 1401 KB)

Supplementary file2 (PDF 2802 KB)

Supplementary file3 (PDF 2165 KB)

Supplementary file4 (PDF 1840 KB)

Supplementary file5 (PDF 2691 KB)

## Abbreviations

AGS: Astrocyte growth factors
BBB: Blood brain barrier
BSA: Bovine serum albumin
CNS: Central nervous system
DIV: Day in vitro
DLS: Dynamic light scattering
DMEM: Dulbecco’s Modified Eagle’s Medium
DMSO: Dimethyl sulfoxide
D_2_O: Deuterated water
DOPC: 1,2-Dioleoyl-sn-glycero-3-phosphocholine
FBS: Foetal bovine serum
GALC: Galactosylceramidase
GFAP: Glial fibrillary acidic protein
HBSS: Hank’s buffered salt solution
Iba1: Ionized calcium binding adaptor 1
IL: Interleukin
KD: Krabbe’s disease
LCN: Lecithin/chitosan nanoparticles
MBP: Myelin basic protein
MOG: Myelin oligodendrocyte glycoprotein
NFH: Neurofilament H
n.s.: Non-significant
overnight: Overnight
P/S: Penicillin/streptomycin
P10: Postnatal day 10
PALS: Phase analysis light Scattering
PBS: Phosphate-buffered saline
PLA2: Phospholipase A2
PDI: Polydispersity Index
Psy: Psychosine
RM: Repeated measures
RT: Room temperature
S1P: Sphingosine-1-phosphate
SEM: Standard error of the mean
TNF-α: Tumour necrosis factor-alpha
WMT: White matter tracts
